# Prevalence of antimicrobial resistance and virulence genes in *Klebsiella pneumoniae* and Congenetic *Raoultella* Isolates from captive giant pandas

**DOI:** 10.1371/journal.pone.0283738

**Published:** 2023-03-30

**Authors:** Yan Li, Yang Sun, Shi-wen Sun, Bing Liang, Bo-wen Jiang, Na Feng, Jun Liu, Xue Ji

**Affiliations:** 1 Engineering Research Center of Glycoconjugates, Ministry of Education, School of Life Sciences, Northeast Normal University, Changchun, China; 2 Changchun Veterinary Research Institute, Chinese Academy of Agricultural Sciences, Changchun, China; 3 Key Laboratory of Jilin Province for Zoonosis Prevention and Control, Changchun, China; University of Iowa, Carver College of Medicine, UNITED STATES

## Abstract

To study antimicrobial resistance and virulence genes of *Klebsiella pneumoniae* and *Raoultella* strains isolated from captive giant pandas. Non-duplicate fecal samples were collected from 128 giant pandas during 2017–2019. All isolated microbial strains were tested for antimicrobial drug susceptibility using BD verification panels. Four extended-spectrum β-lactamase resistance genes, nine virulence genes and six capsular serotype genes were detected using PCR. 42 *K*. *pneumoniae* and nine *Raoultella* strains were isolated from different giant pandas. Antibiotic resistance rates were 1.9%–23.5%, except for ampicillin, and 7.8% of the isolates were multidrug-resistant to 7–10 antibiotic classes. This is the first time that a multidrug-resistant *R*. *ornithinolytica* strain has been isolated from captive giant pandas. The *bla*_TEM_, *bla*_CTX-M_, *bla*_SHV_ and *bla*_DHA_ genes were detected in four MDR ESBL*- K*. *pneumoniae* strains. The *rmpA*, *iutA*, *ybtS*, *iroN* and *iroB* genes were positively detected in 11.7% of the isolates. Capsular serotype (K2, K5, K54 and K57) genes were all detected in four *K*. *pneumoniae* strains, and one was identified as hypervirulent. This study showed that MDR ESBL- *K*. *pneumoniae*, hypervirulent *K*. *pneumoniae*, MDR *R*. *ornithinolytica* and the colistin-resistant strain may pose risks to captive giant pandas and their keepers, and that the diversity of antibiotic resistance and virulence genes in *Klebsiella* and *Raoultella* should be monitored regularly.

## Introduction

*Klebsiella* and *Raoultella* are Gram-negative, facultatively anaerobic, rod-shaped enterobacteria that are ubiquitous in nature (e.g., in water and the soil). As opportunistic pathogens, *Klebsiella* and *Raoultella* can also colonize the mucosal surfaces of humans and other animals, especially in the intestinal tract, and can cause different infections in immunocompromised groups [1,2].

In recent years, the percentage occurrence of *K*. *pneumoniae* in humans has increased from 14% in 2005 to 19.5% in 2017, and become the second most common clinical Gram-negative bacteria after *E*. *coli*, causing community-acquired and nosocomial infections [3,4]. Due to the overuse of antimicrobial drugs and horizontal transfer of resistance genes between species, the emergence of antimicrobial resistance in *K*. *pneumoniae* has become a global public health concern. In 2016, Chakraborty et al. reported that 50% of *K*. *pneumoniae* isolates from clinical samples showed multidrug-resistance (MDR) [5]. Garcia-Fierro R et al. demonstrated the emergence of convergent hypervirulent and MDR *K*. *pneumoniae* (MDR-KP) in animals [6]. Infections caused by hypervirulent *K*. *pneumoniae* (hvKP) are often associated with high morbidity and mortality rates due to their invasiveness and virulence, and can cause serious community-acquired infections in young healthy individuals [7]. The increased prevalence of MDR-KP and hvKP strains will become major public health problems and pose potential threats to the health of humans and animals.

*Raoultella* was initially included in the genus *Klebsiella*, but was later reclassified based on its 16S rDNA sequence and the presence of the *rpoB* gene [8]. However, whether to unify or split *Klebsiella* and *Raoultella* remains a matter of debate. *Raoultella* is also considered a potentially important pathogen. Like *Klebsiella*, *Raoultella* can acquire antibiotic resistance genes from other bacteria, leading to an increase in the occurrence of MDR strains (e.g., extended spectrum beta-lactamase (ESBL) producers), and shares similar virulence factors (e.g., polysaccharide capsules, siderophores) with *Klebsiella* [2,9,10]. *R*. *ornithinolytica* is one of the most frequently encountered *Raoultella* pathogens in humans, and can cause pneumonia, gastrointestinal infections, urinary infections and bacteraemia. The prevalence of *R*. *ornithinolytica* related infections in humans and animals is poorly reported in the literature, possibly due to its misidentification as a *Klebsiella* species in clinical laboratories using conventional phenotypic methods. Matrix-assisted laser desorption/ionization time-of-flight mass spectrometry is an advanced technique that has been used to identify *R*. *ornithinolytica* in human infections. One study showed that the detection and infection rates of *R*. *ornithinolytica* have been increasing, and many MDR strains are increasingly reported year on year [11,12].

As one of the world’s rarest and most familiar animals, the giant panda (*Ailuropoda melanoleuca*) is an important “flagship” species for biodiversity conservation, both in China and worldwide. Previously listed as endangered, the giant panda was recategorized as vulnerable in the 2016 update of *The IUCN Red List of Threatened Species* (https://www.iucnredlist.org/species/712/121745669). Antibacterial drugs have proved to be an effective method to prevent and control infectious diseases in giant pandas, but because of long-term artificial feeding and the wide use of antibiotics, captive giant pandas have become predisposed to microbiota dysbiosis, leading to the emergence of antibiotic resistant bacteria and even MDR bacteria [13,14]. Studies have shown that virulence genes and antibiotic resistance genes enriched in the microbiomes of captive giant pandas were higher than those in wild giant pandas [15]. Zhang AY et al. found that MDR was common in 59 isolates collected from the fecal samples of 30 giant pandas [16]. In 2021, Su et al. found that *K*. *pneumoniae* was common in the gut of captive giant pandas, and that ESBL-producing *K*. *pneumoniae* (ESBL-KP) also occurred (1.42%, 3/211) [17]. One study found that a carbapenem resistant *K*. *pneumoniae* strain isolated from giant pandas was strongly pathogenic in mice [18]. MDR-KP or hvKP infections pose serious threats to the life and health of giant pandas, as well as posing a potential threat to panda breeders and even to wildlife center visitors. However, there have so far been no reports of giant panda infection with *R*. *ornithinolytica*.

This study aimed to investigate the characteristics of antimicrobial resistance and virulence of *K*. *pneumoniae* and *R*. *ornithinolytica* strains isolated from captive giant pandas, to improve our baseline data on the two bacteria and help to prevent the spread of MDR, hvKP and *R*. *ornithinolytica* strains.

## Materials and methods

### Isolation and identification of *K*. *pneumoniae* and *R*. *ornithinolytica* strains

Fecal samples were collected from 128 captive giant pandas at three giant panda breeding facilities (Dujiangyan Base, Ya’an Bifengxia Base and Chengdu Base) in Sichuan Province, China. All samples were kept at low temperature for transport to the laboratory. The samples were resuspended in physiological saline, plated on CHROMagar™ Shigella Agar (Chromagar, Paris, France) and incubated overnight at 37°C. One sample bacterial colony was selected from each plate for subsequent analysis. The initial identification of isolates was determined using the NMIC/ID 4 panel of a BD Phoenix™ Automated Identification and Susceptibility Testing System (Becton, Dickinson and Company, Franklin Lakes, NJ, USA). Specific genes (*rpoB*, *pehX* and *bla*_ORN_) were used to further identify the *K*. *pneumoniae* and *R*. *ornithinolytica* strains (Table 1) [19–21]. The PCR reaction mixture contained 12.5 μl 2×Taq DNA Master Mix (CWBio, Beijing, China), 0.5 μl of each primer at a concentration of 10 μm, 1 μl of the DNA template, made up to a final volume of 25 μl with ddH_2_O. The details are shown (Table 1).

**Table 1:** Primers used in the study.

Gene	Primers	Sequences (5’-3’)	Product (bp)	Reference
*rpoB*	rpoB-F	CAACGGTGTGGTTACTGACG	108	[19]
	rpoB-R	TCTACGAAGTGGCCGTTTTC		
*pehX*	pehX-F	GATACGGAGTATGCCTTTACGGTG	343	[20]
	pehX-R	ACCGTTGATCACTTCGGTCAGG		
*bla*_ORN_	VO1	CATACCAGCCTGAATGAA	1128	[21]
	VO2	GTTTTGTCCGGGGATGTT		
*bla*_OXA_	OXA-F	GGCACCAGATTCAACTTTCAAG	564	[22]
	OXA-R	GACCCCAAGTTTCCTGTAAGTG		
*bla*_TEM_	TEM-F	ATGAGTATTCAACATTTCCGT	861	[23]
	TEM-R	TTACCAATGCTTAATCAGTGA		
*bla*_CTX-M_	CTX-MU1	ATGTGCAGYACCAGTAARGT	544	[24]
	CTX-MU2	TGGGTRAARTARGTSACCAGA		
*bla*_SHV_	SHV-F	ATTTGTCGCTTCTTTACTCGC	1018	[24]
	SHV-R	TTTATGGCGTTACCTTTGACC		
*wzy-K1*(*magA*)	K1-F	GGTGCTCTTTACATCATTGC	1283	[25]
	K1-R	GCAATGGCCATTTGCGTTAG		
*wzy-K2*	K2-F	GACCCGATATTCATACTTGACAGAG	641	[25]
	K2-R	CCTGAAGTAAAATCGTAAATAGAT		
*wzx-K5*	K5-F	TGGTAGTGATGCTCGCGA	280	[25]
	K5-R	CCTGAACCCACCCCAATC		
*wzy-K20*	K20-F	CGGTGCTACAGTGCATCATT	741	[26]
	K20-R	GTTATACGATGCTCAGTCGC		
*wzx-K54*	K54-F	CATTAGCTCAGTGGTTGGCT	881	[26]
	K54-R	GCTTGACAAACACCATAGCAG		
*wzy-K57*	K57-F	CTCAGGGCTAGAAGTGTCAT	1037	[26]
	K57-R	CACTAACCCAGAAAGTCGAG		
*rmpA*	rmpA-F	ACTGGGCTACCTCTGCTTCA	535	[27]
	rmpA-R	CTTGCATGAGCCATCTTTCA		
*wcaG*	wcaG-F	GGTTGGKTCAGCAATCGTA	169	[28]
	wcaG-R	ACTATTCCGCCAACTTTTGC		
*rmpA2*	rmpA2-F	GTGCAATAAGGATGTTACATTA	430	[29]
	rmpA2-R	GGATGCCCTCCTCCTG		
*iucA*	iucA-F	AATCAATGGCTATTCCCGCTG	239	[29]
	iucA-R	CGCTTCACTTCTTTCACTGACAGG		
*iroB*	iroB-F	ATCTCATCATCTACCCTCCGCTC	235	[29]
	iroB-R	GGTTCGCCGTCGTTTTCAA		
*peg-344*	peg-344-F	CTTGAAACTATCCCTCCAGTC	508	[29]
	peg-344-R	CCAGCGAAAGAATAACCCC		
*iutA*	iutA-F	GGCTGGACATCATGGGAACTGG	300	[30]
	iutA-R	CGTCGGGAACGGGTAGAATCG		
*ybtS*	ybtS-F	AGTGGTGCGTTCTGCGTC	477	[30]
	ybtS-R	ATTTCTACATCTGGCGTTA		
*iroN*	iroN-F	CACGACCAATCGCCTGAG	440	In this study
	iroN-R	CGTTGCCTTTCGAGTAGAGC		

### Antimicrobial susceptibility test

The susceptibilities of all isolates to 21 antimicrobials (in 13 categories) were tested using the NMIC/ID 4 panel of a BD Phoenix™ Automated Identification and Susceptibility Testing System. The antibiotics tested included amikacin (AMK), aztreonam (ATM), ampicillin (AMP), amoxicillin-clavulanate (AMC), cefazolin (CFZ), ceftazidime (CAZ), cefotaxime (CTX), colistin (CST), chloramphenicol (CHL), ciprofloxacin (CIP), cefepime (FEP), gentamicin (GEN), imipenem (IPM), levofloxacin (LVX), meropenem (MEM), moxifloxacin (MXF), piperacillin (PIP), ampicillin-Sulbactam (SAM), trimethoprim-sulfamethoxazole (SXT), piperacillin-tazobactam (TZP) and tetracycline (TET). The results were interpreted in accordance with the Clinical and Laboratory Standards Institute criteria of 2020. MDR isolates were defined as those isolates resistant to ≥3 antimicrobial classes in addition to ampicillin, which is known to be intrinsically resistant for *Klebsiella* and *Raoultella* [31–33]. The test system also measured the ESBL phenotypes.

### Determination of the minimum inhibitory concentration (MIC)

The NMIC/ID 4 panel of the BD Phoenix™ Automated Identification and Susceptibility Testing System has low accuracy in distinguishing colistin-resistant and colistin-susceptible strains [34]. Based on the identification results of the BD Phoenix™ system, the minimum inhibitory concentration was determined using the broth microdilution method to obtain a more definitive identification of colistin-resistant strains. The MIC was tested using cation-adjusted Mueller-Hinton broth. The broth microdilution method was performed in accordance with the 2020 Clinical and Laboratory Standards Institute guidelines. *E*. *coli* ATCC 25922 was used as a control strain.

### Detection of ESBL genes using PCR

A DNA template consisting of boiled lysates was prepared from the isolates. The strains with ESBL phenotypes were screened for the presence of ESBL genes (*bla*_OXA_, *bla*_TEM_, *bla*_CTX-M_ and *bla*_SHV_) using PCR. The PCR reaction mixture contained 12.5 μl 2×Taq DNA Master Mix (CWBio, Beijing, China), 0.5 μl of each primer at a concentration of 10 μm, 1 μl of the DNA template, and made up to a final volume of 25 μl with ddH_2_O. The details are shown (Table 1). PCR products were sent to Kumei Biotech (Jilin, China) for sequencing. The sequencing data were analyzed using the BLAST search engine (http://blast.ncbi.nlm.nih.gov/Blast.cgi).

### Modified string test

The string test, where a bacteriology inoculation loop is used to stretch a mucoviscous string from a bacterial colony, was used to evaluate mucoid phenotypes [35]. The strains were inoculated on 5% sheep blood plates and incubated at 37°C overnight. A positive modified string test was recorded when a mucoviscous string formed (length >10 mm) when an inoculation loop was used to stretch a colony on the plate, thus indicating that the strain had a hypermucoviscosity phenotype [36].

### Detection of virulence-related genes using PCR

Capsular serotype-specific genes, including six capsular serotypes (K1, K2, K5, K20, K54, K57) and virulence genes, were detected using PCR. The virulence genes found were: capsular polysaccharide synthesis regulators (the plasmid genes *rmpA* and *rmpA2*); the capsule associated gene (*wcaG*); iron acquisition system genes (*iutA*, *iucA*, *iroB*, *iroN* and *ybtS*); and a metabolic transporter gene (*peg-344*).The primers and amplification conditions used followed practices established in the literature [25–30]. The primers used are listed (Table 1). The PCR products were sequenced by Kumei Biotech (Jilin, China). The sequencing data were analyzed using the BLAST search engine (http://blast.ncbi.nlm.nih.gov/Blast.cgi).

### Short-read whole-genome sequencing

Isolates with either the ESBL phenotype, colistin-resistant phenotype, or virulence associated genes were selected for sequencing. The genomic DNA of the isolates was extracted using a bacterial DNA extraction kit (Omega Bio-Tek Company, Norcross, GA, USA). The harvested DNA was detected using agarose gel electrophoresis and quantified using a Qubit® 2.0 Fluorometer (Thermo Scientific, Waltham, MA, USA). The whole genome of each isolate was sequenced using an Illumina NovaSeq PE150 at the Beijing Novogene Bioinformatics Technology Co., Ltd. (Beijing, China). Predictions of virulence genes and antimicrobial resistance genes were based on the VFDB (Virulence Factors of Pathogenic Bacteria; http://www.mgc.ac.cn/cgi-bin/VFs) and CARD (Comprehensive Antibiotic Research Database; fhttps://card.mcmaster.ca/) databases.

## Results

### Isolation of strains

A total of 42 (82.3%) *K*. *pneumoniae* and nine (17.6%) *Raoultella* isolates were extracted from 128 giant panda feces collected at three breeding facilities in Sichuan Province, China. Based on the results of a BD Phoenix system, PCR analysis, and short-read whole-genome sequencing, the *Raoultella* strains included eight *R*. *ornithinolytica* and one *Raoultella* strain. Each nonduplicated strain was isolated from a single giant panda fecal sample. The isolation rate of strains in Dujiangyan Base, Ya’an Bifengxia Base and Chengdu Base were 25.0% (7/28), 75.0% (12/16) and 38.1% (32/84), respectively.

### Antimicrobial resistance analysis

The 51 strains showed different antibiotic resistance spectra to 21 antimicrobials (Table 2): 42 (82.3%) of the isolates were resistant to one or more of 15 different antibiotics, with 78.4%, 23.5%, 17.6%, 13.7%, 13.7%, and 11.7% being resistant to ampicillin, tetracycline, trimethoprim-sulfamethoxazole, piperacillin, chloramphenicol, and ampicillin-sulbactam, respectively. Furthermore, fewer than 7.8% of the isolates were resistant to either gentamicin, cefazolin, cefotaxime, cefepime, aztreonam, ciprofloxacin, ceftazidime, amoxicillin-clavulanate, or colistin. All of the isolates were sensitive to amikacin, imipenem, meropenem, piperacillin-tazobactam, levofloxacin, and moxifloxacin.

**Table 2:** Antimicrobial resistance of strains.

Antimicrobials	Isolates (n = 51)	MDR isolates (n = 10)	ESBL-KP isolates(n = 4)
AMK	-	-	-
GEN	4(7.8%)	4(40.0%)	3(75.0%)
IPM	-	-	-
MEN	-	-	-
CFZ	4(7.8%)	4(40.0%)	4(100.0%)
CAZ	1(1.9%)	1(10.0%)	1(25.0%)
CTX	3(5.8%)	3(30.0%)	3(75.0%)
FEP	3(5.8%)	3(30.0%)	3(75.0%)
ATM	2(3.9%)	2(20.0%)	2(50.0%)
AMP	40(78.4%)	10(100.0%)	4(100.0%)
PIP	7(13.7%)	7(70.0%)	4(100.0%)
AMC	1(1.9%)	1(10.0%)	1(25.0%)
SAM	6(11.7%)	5(50.0%)	4(100.0%)
TZP	-	-	-
CST	1(1.9%)	-	-
SXT	9(17.6%)	9(90.0%)	4(100.0%)
CHL	7(13.7%)	7(70.0%)	3(75.0%)
CIP	2(3.9%)	2(20.0%)	2(50.0%)
LVX	-	-	-
MXF	-	-	-
TET	12(23.5%)	9(90.0%)	3(75.0%)

AMK, amikacin; ATM, aztreonam; AMP, ampicillin; AMC, amoxicillin-clavulanate; CFZ, cefazolin; CAZ, ceftazidime; CTX, cefotaxime; CST, colistin; CHL, chloramphenicol; CIP, ciprofloxacin; FEP, cefepime; GEN, gentamicin; IPM, imipenem; LVX, levofloxacin; MEN, meropenem; MXF, moxifloxacin; PIP, piperacillin; SXT, trimethoprim-sulfamethoxazole; SAM, ampicillin-sulbactam; TZP, piperacillin-tazobactam; TET, tetracycline.

Among the 42 resistant strains, 10 (23.8%) were resistant to three or more different classes of antimicrobials (i.e., were an MDR strain) (Table 3). The MDR isolates included nine *K*. *pneumoniae* and one *R*. *ornithinolytica*. The most common MDR phenotype was ampicillin- trimethoprim-sulfamethoxazole-chloramphenicol-tetracycline (2/10, 20.0%). Among the nine MDR-KP isolates, four (44.4%) were also ESBL producers that were resistant to 7–10 classes of antibiotics (Table 3).

**Table 3:** Resistance patterns of MDR isolates.

Isolate	Species	Resistance patterns
16	*K*. *pneumoniae*	AMP, PIP, SXT, CHL, TET
4*	*K*. *pneumoniae*	GEN, CFZ, CTX, FEP, ATM, AMP, PIP, SAM, SXT, CHL, CIP, TET
13*	*K*. *pneumoniae*	GEN, CFZ, CTX, FEP, AMP, PIP, SAM, SXT, CHL, CIP, TET
44	*K*. *pneumoniae*	AMP, PIP, SAM, TET
KP-30	*K*. *pneumoniae*	GEN, AMP, SXT, CHL, TET
KP-41-1	*K*. *pneumoniae*	AMP, SXT, CHL, TET
KP-53-1	*K*. *pneumoniae*	AMP, SXT, CHL, TET
KP-54-1*	*K*. *pneumoniae*	CFZ, CTX, FEP, ATM, AMP, PIP, SAM, SXT, TET
KP-PD42*	*K*. *pneumoniae*	GEN, CFZ, CAZ, AMP, PIP, AMC, SAM, SXT, CHL
KP-PD111	*R*. *ornithinolytica*	AMP, PIP, SXT, TET

ATM, aztreonam; AMP, ampicillin; AMC, amoxicillin-clavulanate; CFZ, cefazolin; CAZ, ceftazidime; CTX, cefotaxime; CHL, chloramphenicol; CIP, ciprofloxacin; FEP, cefepime; GEN, gentamicin; PIP, piperacillin; SXT, trimethoprim-sulfamethoxazole; SAM, ampicillin-sulbactam; TET, tetracycline.

*ESBL-producing isolates.

### MIC of colistin

Only the KP-43 isolate had a colistin resistant phenotype. The results of the three broth microdilution method replicates showed that the MIC of the colistin > 32 μg/ml. The KP-43 strain was therefore resistant to colistin according to the 2020 Clinical and Laboratory Standards Institute guidelines.

### ESBL genes of ESBL-producing isolates

The PCR and short-read whole-genome sequencing results showed that the beta-lactam resistance genes of the ESBL-KP strains found in this study generally showed mixed genotype characteristics (Table 4). Four ESBL-KP isolates harbored *bla*_TEM-1_, two harbored *bla*_CTX-M-3_ and one harbored *bla*_CTX-M-15_. Four kinds of *bla*_SHV_ genes (*bla*_SHV-27_, *bla*_SHV-28_, *bla*_SHV-81_ and *bla*_SHV-193_) were also identified. In addition, the *bla*_DHA-1_ gene was detected in one strain. The *bla*_OXA_ gene was not detected in any of the isolates.

**Table 4:** The beta-lactam resistance genes of four ESBL-KP isolates.

Isolate	beta-lactam resistance genes
4	*bla*_TEM-1_, *bla*_CTX-M-3_, *bla*_SHV-81_
13	*bla*_TEM-1_, *bla*_CTX-M-3_, *bla*_SHV-193_
KP-54-1	*bla*_TEM-1_, *bla*_CTX-M-15_, *bla*_SHV-28_
KP-PD42	*bla*_TEM-1_, *bla*_SHV-27_, *bla*_DHA-1_

### Detection of the hypermucoviscosity phenotype and virulence-related genes

Three strains could form a mucoviscous string > 10 mm in length, including two *K*. *pneumoniae* and one *R*. *ornithinolytica*. These strains were identified as hypermucoviscous (HMV) strains. A diagram of the modified string test is shown in the (S1 Fig). The PCR and short-read whole-genome sequencing results showed five kinds of virulence gene (*rmpA*, *iutA*, *ybtS*, *iroN* and *iroB*) detected in six isolates, either singly or in multiples. The detection rate of the *ybtS* gene was highest (9.8%) and the other genes (*rmpA*, *iroN*, *iroB* and *iutA*) were very low (1.9%-3.9%), four genes (*rmpA2*, *wcaG*, *iucA* and *peg-344*) were not detected in any of the isolates (Table 5). Four kinds of capsular serotypes (K2, K5, K54 and K57) were detected in four *K*. *pneumoniae* strains, and only one of the *K*. *pneumoniae* strains carried virulence genes (Table 6). Only one HMV KP strain carried both the *rmpA* and the K5 capsular serotype genes, and was defined as hvKP. The details are shown (Table 6).

**Table 5:** The isolation rate of virulence-related genes.

Virulence-related genes		Isolation rate (%)
virulence genes	*rmpA*	1(1.9%)
	*rmpA2*	-
	*wcaG*	-
	*iutA*	2(3.9%)
	*iucA*	-
	*iroB*	1(1.9%)
	*iroN*	1(1.9%)
	*ybtS*	5(9.8%)
	*peg-344*	-
Capsular serotype	*K1(magA)*	-
	*K2*	1(1.9%)
	*K5*	1(1.9%)
	*K20*	-
	*K54*	1(1.9%)
	*K57*	1(1.9%)

**Table 6:** The results of hypermucoviscosity phenotype and virulence-related genes.

Isolate	Species	Virulence genes	Capsular serotype	Modified string test
29	*K*. *pneumoniae*	*iutA*, *ybtS*	ND	Negative
41	*K*. *pneumoniae*	*rmpA*, *iroN*, *iroB*, *ybtS*	K5	Positive
KP-22	*K*. *pneumoniae*	*ybtS*	ND	Negative
KP-28	*K*. *pneumoniae*	ND	K57	Negative
KP-35	*K*. *pneumoniae*	ND	K54	Negative
KP-41-1	*K*. *pneumoniae*	*iutA*	ND	Negative
KP-54-1	*K*. *pneumoniae*	ND	K2	Negative
KP-60	*K*. *pneumoniae*	ND	ND	Positive
KP-43	*Raoultella*	*ybtS*	ND	Negative
KP-PD111	*R*. *ornithinolytica*	*ybtS*	ND	Positive

ND, Not detected.

## Discussion

In 2020, Yan X et al. obtained the first isolates of MDR-KP in 16.5% (30/182) from the feces of healthy captive giant pandas [37]. In the present study, 42 *K*. *pneumoniae* strains were isolated from the feces of clinical healthy captive giant pandas, with an isolation rate of MDR-KP of 21.4% (9/42), a little higher than Yan X et al. Our higher isolation rate of MDR-KP strains may be due to differences in the ecological environment of the breeding facilities, and the degree of contact between giant pandas and tourists. This is the first report of *R*. *ornithinolytica* strains (15.6%, 8/51) having been isolated from captive giant pandas, including an MDR *R*. *ornithinolytica* strain (KP-PD111). *R*. *ornithinolytica* strains are often resistant to aminoglycosides, quinolones and sulfonamides, and the gene responsible for resistance is located on a plasmid [2]. KP-PD111 was also resistant to penicillin, trimethoprim-sulfamethoxazole, and tetracycline, so might contain a resistant plasmid. Due to the close relationship between the genera *Raoultella* and *Klebsiella*, plasmid-mediated horizontal transfer of resistance genes may lead to the emergence of more MDR *Raoultella* or MDR *Klebsiella* strains in the future. KP-PD111 had the hypermucoviscous phenotype but lacked the hypermucoviscous genes (*rmpA* and *rmpA2*), suggesting the existence of novel genetic determinants of hypermucoviscosity. Because *Raoultella* is very similar to *Klebsiella*, some authors have hypothesized that *Raoultella* spp. could play a role as a reservoir of antimicrobial resistance genes [33]. Due to the difficulty in distinguishing between *Klebsiella* and *Raoultella*, the clinical features and antimicrobial susceptibility of *Raoultella* (e.g., *R*. *ornithinolytica*) remain to be properly elucidated. Therefore, it should be of concern that MDR *R*. *ornithinolytica* has now appeared in captive giant pandas, and those breeding giant pandas should adapt their infection management control and prevention measures accordingly. In 2015, China has banned the use of colistin as a growth promoter (as a feed additive) for animals [38]. Colistin is considered to be one of the few therapies for serious infections caused by MDR, especially ESBL-producing strains. This study found that the sensitive KP-43 strain did not carry the colistin-resistant gene, and that the minimum MIC to colistin of this strain > 32 μg/ml. Because this strain may have unknown resistance mechanisms, it is important to conduct epidemiological investigations of colistin-resistant bacteria in giant panda breeding facilities.

Chiaverini A et al. isolated 16 *K*. *pneumoniae* strains from wild animals (15 mammals and one bird), of which 56.2% (9/16) were classified as MDRs, with rates of resistance to tetracycline, trimethoprim and β-lactams of 18.8%–100.0% [39]. The reason for the low isolation rates of MDR strains in this study may be because captive giant pandas do not live in groups so bacterial spread between individuals is low, or it may be because of their better habitat.

While some studies have claimed that chloramphenicol and tetracycline have not been used as therapeutic drugs in giant pandas [16,37], Zhang AY et al. found that 13.56% of the bacterial strains isolated from the feces of 30 giant pandas were resistant to chloramphenicol and tetracycline [16]. Yan X et al. found resistance rates of 30 MDR-KP strains to chloramphenicol and tetracycline of 60% [37]. Interestingly, the MDR strains found in this study showed slightly higher resistance to chloramphenicol (70%) and trimethoprim-sulfamethoxazole (90%). Antimicrobial residues from different sources in the environment and the horizontal transmission of antimicrobial resistance genes have far-reaching impacts on the antimicrobial resistance phenotypes of bacteria. In addition, some isolates have been found to be intermediately sensitive to several antibiotics, including some β-lactam and quinolone antibiotics. A survey on the use of antibiotics in giant pandas indicated that β-lactam antibiotics account for about 50% of antibiotic use, including carbapenem antibiotics such as imipenem [40]. In this study, all of the isolates were sensitive to carbapenem antibiotics (imipenem and meropenem), and had low resistance rates to cefepime, cefotaxime, cefazolin, ampicillin-sulbactam, and piperacillin (5.8%–13.7%). However, among four ESBL-KP isolates, 25.0%–100.0% were resistant to nine β-lactam antibiotics other than carbapenems antibiotics and piperacillin-tazobactam. Sun X et al. identified three (1.42%) ESBL-KPs from 211 nonduplicated *K*. *pneumoniae* isolates collected from the fresh feces of captive giant pandas [17]. In contrast, the isolation of MDR ESBL-KP strains was higher (7.8%, 4/51) in this study. This difference might be associated with differences in the giant pandas’ living conditions and the choice of antibiotics tested. ESBL-producing *Enterobacteriaceae* (e.g., *K*. *pneumoniae*) are important human AROs and are common causes of urinary tract and bloodstream infections [41]. Based on World Health Organization reports, *K*. *pneumoniae* strains with ESBL has reached a 50% prevalence rate in many parts of the world, with a community resistance rate of 30%, indicating the ubiquity of this resistance [42]. MDR strains with ESBL phenotypes are reported to be associated with higher morbidity and mortality rates [43,44]. ESBL-producing bacteria present huge challenges to healthcare, owing to the restricted empirical options available for treating infections. There has been increasing use of carbapenems as the last line of effective therapy for infections caused by MDR KP, and this has led to the emergence and global spread of *K*. *pneumoniae* isolates with resistance genes coding for carbapenemases [45,46]. Increasing prevalence of MDR ESBL-KP in giant pandas would limit the choice of clinical antibiotics and may increase the occurrence of carbapenem-resistant strains. Therefore, it is important to use antimicrobials in giant pandas with caution, and regular surveys of antimicrobial resistance in giant panda isolates are needed to achieve the clinically rational use of antibiotics in this species.

One study showed that transmission and adaptation of ESBL-KP can be found in zoo environments [47]. Su X et al. detected ESBL-encoding genes (*bla*_TEM_, *bla*_CTX-M-1_ and *bla*_SHV_) in three ESBL-KP giant panda fecal isolates [17]. Wang X et al. detected the *bla*_CTX-M-3_ and *bla*_TEM-1_ genes in an MDR-KP strain isolated from giant panda feces [48]. In this study, four ESBL-KP strains predominantly contained the *bla*_CTX-M-3_ or *bla*_CTX-M-15_, *bla*_TEM-1_ and *bla*_SHV_ genes. We also found that two MDR ESBL-KP strains carried both the *bla*_CTX-M-3_ and *bla*_TEM-1_ genes. Over the past decade, ESBLs of the CTX-M type have been recognized to be of growing importance worldwide and are increasingly widely reported among enterobacterial isolates, mainly of *Escherichia*. *coli* and *K*. *pneumoniae* [49]. *bla*_CTX-M-15_ is currently the *bla*_CTX-M_ variant most commonly identified in enterobacterial species of human origin worldwide [50]. Importantly, the *bla*_TEM_ and *bla*_CTX-M_ genes, which have been connected with mobile genetic elements such as transposons and plasmids, have been shown to cause horizontal gene spread between the same or different species of populations of Gram-negative bacteria [51,52]. Our sequencing and comparison procedures found no colistin resistance genes (*mcr-1* and *mcr-2*) in the colistin-resistant strain (KP-43). This indicates that other resistance mechanisms might be responsible for this high level of colistin resistance. The *bla*_DHA-1_ gene was detected in an MDR ESBL-KP strain (KP-PD42). The plasmid-encoded AmpC-type β-lactamase genes (such as *bla*_DHA_) have been recognized to be of growing importance and have been reported globally from human and animal isolates [53,54]. Therefore, it is necessary to monitor the emergence and spread of ESBL and AmpC genes in ESBL-KP in captive giant pandas, and between human and giant panda bacterial strains.

We found three HMV *K*. *pneumoniae* strains using a modified string test, only one of which carried four virulence genes. Some studies have tended to use a combination of the hypermucoviscosity phenotype, capsular serotype (K1 and K2) and key virulence genes (e.g., *rmpA*, *rmpA2*, *iucA*, and *iroB*, etc.) to define hvKP [55–58]. Currently 77 capsular serotypes of *K*. *pneumoniae* are known, of which serotype K5 is one of those associated with severe invasive infections in humans [28]. In our study, we detected four virulence genes (*rmpA*, *iroN*, *iroB* and *ybtS*) from a K5 type HMV *K*. *pneumoniae* strain preliminarily defined as hvKP. In African green monkeys (*Chlorocebus aethiops sabaeus*), serotypes K5 have been implicated in fatal multisystemic abscesses [59]. Therefore, the hvKP found in this study is potentially pathogenic to giant pandas and should be a priority for treatment.

## Conclusions

In conclusion, we found that antimicrobial resistance was widely distributed among *K*. *pneumoniae* strains and an *R*. *ornithinolytica* strain isolated from fecal samples taken from healthy captive giant pandas, especially ESBL and colistin resistance. We also found an hvKP strain with serotype K5, which was susceptible to 20 antibiotics (except AMP). The spread of the hvKP strain, the colistin-resistant strain and MDR strains (especially MDR ESBL-KP) will pose serious health risks to captive giant pandas, and also to their breeders. The horizontal transfer of resistance or virulence genes located in plasmids and other genetic elements between MDR and hypervirulent strains could produce an MDR hypervirulent strain resulting in high mortality. Consequently, it is essential that relevant precautionary measures are taken to prevent this risk. Considering the specific living conditions and habits of captive giant pandas, as well as their daily medication, their clinical treatment needs to be standardized by the development of stricter guidelines for the clinical use of antibiotics. We recommend paying close attention to the selection of antimicrobials and epidemiological surveillance in captive giant pandas to prevent future outbreaks of *Klebsiella* and *Raoultella* infections in their populations.

## Supporting information

S1 Fig The diagram of the modified string test. (TIF)

S1 Table The sequences of beta-lactam resistance genes of four ESBL-producing isolates. (DOCX)

S2 Table The sequences of the virulence-related genes. (DOCX)
