# Supplementary figures and images for: Prevalence of antimicrobial resistance and virulence genes in Klebsiella pneumoniae and Congenetic Raoultella Isolates from captive giant pandas
Source: PLoS One. 2023 Mar 30;18(3):e0283738. doi: 10.1371/journal.pone.0283738 (PMC10062605; doi:10.1371/journal.pone.0283738)

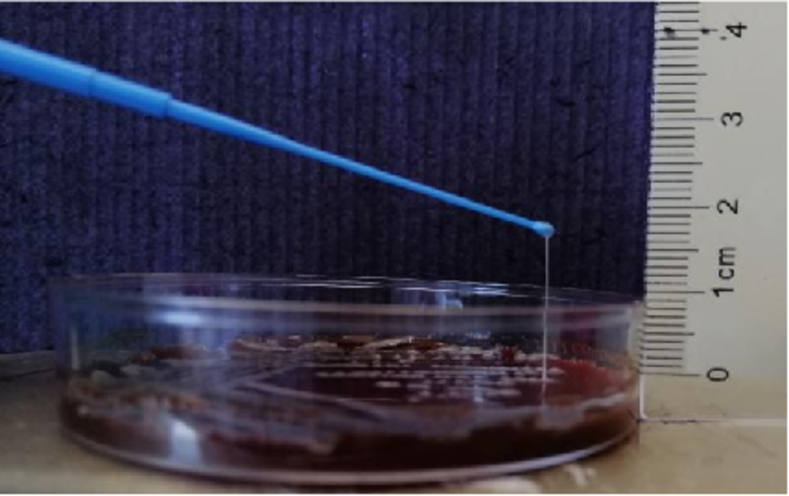

Supplement: S1 Fig — (TIF) [file pone.0283738.s001.tif]
