# Supplementary material for: Prevalence of antimicrobial resistance and virulence genes in Klebsiella pneumoniae and Congenetic Raoultella Isolates from captive giant pandas
Source: PLoS One. 2023 Mar 30;18(3):e0283738. doi: 10.1371/journal.pone.0283738 (PMC10062605; doi:10.1371/journal.pone.0283738)
Supplement: S1 Table — (DOCX) [file pone.0283738.s002.docx]

**S1 Table. The sequences of beta-lactam resistance genes of four ESBL-producing isolates.**

| **Isolate** | **gene** | **Sequence** | **Reference sequence**  **(accession number)** | **Homology**  **(%)** |
| --- | --- | --- | --- | --- |
| 4 | *bla*_CTX-M-3_ | GGCATGAGGCGCGGCGCGGTGCTGAGAAGTGAAAGCGAACCGAATCTGTTAAATCAGCGAGTTGAGATCAAAAAATCTGACCTTGTTAACTATAATCCGATTGCGGAAAAGCACGTCAATGGGACGATGTCACTGGCTGAGCTTAGCGCGGCCGCGCTACAGTACAGCGATAACGTGGCGATGAATAAGCTGATTGCTCACGTTGGCGGCCCGGCTAGCGTCACCGCGTTCGCCCGACAGCTGGGAGACGAAACGTTCCGTCTCGACCGTACCGAGCCGACGTTAAACACCGCCATTCCGGGCGATCCGCGTGATACCACTTCACCTCGGGCAATGGCGCAAACTCTGCGGAATCTGACGCTGGGTAAAGCATTGGGCGACAGCCAACGGGCGCAGCTGGTGACATGGATGAAAGGCAATACCACCGGTGCAGCGAGCATTCAGGCTGGACTGCCTGCTTCCTGGGTTGTGGGGGATAAAACCGGCAGCGGTGACTATGGCACCACCAACGTAATTCGCGGTCAG | Y10278 | 100.00 |
|  | *bla*_TEM-1_ | ATGAGTATTCAACATTTTCGTGTCGCCCTTATTCCCTTTTTTGCGGCATTTTGCCTTCCTGTTTTTGCTCACCCAGAAACGCTGGTGAAAGTAAAAGATGCTGAAGATCAGTTGGGTGCACGAGTGGGTTACATCGAACTGGATCTCAACAGCGGTAAGATCCTTGAGAGTTTTCGCCCCGAAGAACGTTTTCCAATGATGAGCACTTTTAAAGTTCTGCTATGTGGTGCGGTATTATCCCGTGTTGACGCCGGGCAAGAGCAACTCGGTCGCCGCATACACTATTCTCAGAATGACTTGGTTGAGTACTCACCAGTCACAGAAAAGCATCTTACGGATGGCATGACAGTAAGAGAATTATGCAGTGCTGCCATAACCATGAGTGATAACACTGCTGCCAACTTACTTCTGACAACGATCGGAGGACCGAAGGAGCTAACCGCTTTTTTGCACAACATGGGGGATCATGTAACTCGCCTTGATCGTTGGGAACCGGAGCTGAATGAAGCCATACCAAACGACGAGCGTGACACCACGATGCCTGCAGCAATGGCAACAACGTTGCGCAAACTATTAACTGGCGAACTACTTACTCTAGCTTCCCGGCAACAATTAATAGACTGGATGGAGGCGGATAAAGTTGCAGGACCACTTCTGCGCTCGGCCCTTCCGGCTGGCTGGTTTATTGCTGATAAATCTGGAGCCGGTGAGCGTGGGTCTCGCGGTATCATTGCAGCACTGGGGCCAGATGGTAAGCCCTCCCGTATCGTAGTTATCTACACGACGGGGAGTCAGGCAACTATGGATGAACGAAATAGACAGATCGCTGAGATAGGTGCCTCACTGATTAAGCATTGGTAA | AY458016 | 100.00 |
|  | *bla*_SHV-81_ | ATGCGTTATATTCGCCTGTGTATTATCTCCCTGTTAGCCACCCTGCCGCTGGCGGTACACGCCAGCCCGCAGCCGCTTGAGCAAATTAAACAAAGCGAAAGCCAGCTGTCGGGCCGCGTAGGCATGATAGAAATGGATCTGGCCAGCGGCCGCACGCTGACCGCCTGGCGCGCCGATGAACGCTTTCCCATGATGAGCACCTTTAAAGTAGTGCTCTGCGGCGCAGTGCTGGCGCGGGTGGATGCCGGTGACGAACAGCTGGAGCGAAAGATCCACTATCGCCAGCAGGATCTGGTGGACTACTCGCCGGTCAGCGAAAAACATCTTGCCGACGGCATGACGGTCGGCGAACTCTGTGCCGCCGCCATTACCATGAGCGATAACAGCGCCGCCAATCTGCTGCTGGCCACCGTCGGCGGCCCCGCAGGATTGACTGCCTTTTTGCGCCAGATCGGCGACAACGTCACCCGCCTTGACCGCTGGGAAACGGAACTGAATGAGGCGCTTCCCGGCGACGCCCGCGACACCACTACCCCGGCCAGCATGGCCGCGACCCTGCGCAAGCTGCTGACCAGCCAGCGTCTGAGCGCCCGTTCGCAACGGCAGCTGCTGCAGTGGATGGTGGACGATCGGGTCGCCGGACCGTTGATCCGCTCCGTGCTGCCGGCGGGCTGGTTTATCGCCGATAAGACCGGAGCTGGCGAGCGGGGTGCGCGCGGGATTGTCGCCCTGCTTGGCCCGAATAACAAAGCAGAGCGCATTGTGGTGATTTATCTGCGGGATACCCCGGCGAGCATGGCCGAGCGAAATCAGCAAATCGCCGGGATCGGCGCGGCGCTGATCGAACACTGGCAACGCTAA | AM176556 | 99.76 |
| 13 | *bla*_CTX-M-3_ | ATGGTTAAAAAATCACTGCGCCAGTTCACGCTGATGGCGACGGCAACCGTCACGCTGTTGTTAGGAAGTGTGCCGCTGTATGCGCAAACGGCGGACGTACAGCAAAAACTTGCCGAATTAGAGCGGCAGTCGGGAGGCAGACTGGGTGTG  GCATTGATTAACACAGCAGATAATTCGCAAATACTTTATCGTGCTGATGAGCGCTTTGCGATGTGCAGCACCAGTAAAGTGATGGCCGCGGCCGCGGTGCTGAAGAAAAGTGAAAGCGAACCGAATCTGTTAAATCAGCGAGTTGAGATCAAAAAATCTGACCTTGTTAACTATAATCCGATTGCGGAAAAGCACGTCAATGGGACGATGTCACTGGCTGAGCTTAGCGCGGCCGCGCTACAGTACAGCGATAACGTGGCGATGAATAAGCTGATTGCTCACGTTGGCGGCCCGGCTAGCGTCACCGCGTTCGCCCGACAGCTGGGAGACGAAACGTTCCGTCTCGACCGTACCGAGCCGACGTTAAACACCGCCATTCCGGGCGATCCGCGTGATACCACTTCACCTCGGGCAATGGCGCAAACTCTGCGGAATCTGACGCTGGGTAAAGCATTGGGCGACAGCCAACGGGCGCAGCTGGTGACATGGATGAAAGGCAATACCACCGGTGCAGCGAGCATTCAGGCTGGACTGCCTGCTTCCTGGGTTGTGGGGGATAAAACCGGCAGCGGTGACTATGGCACCACCAACGATATCGCGGTGATCTGGCCAAAAGATCGTGCGCCGCTGATTCTGGTCACTTACTTCACCCAGCCTCAACCTAAGGCAGAAAGCCGTCGCGATGTATTAGCGTCGGCGGCTAAAATCGTCACCGACGGTTTGTAA | Y10278 | 100.00 |
|  | *bla*_TEM-1_ | ATGAGTATTCAACATTTTCGTGTCGCCCTTATTCCCTTTTTTGCGGCATTTTGCCTTCCTGTTTTTGCTCACCCAGAAACGCTGGTGAAAGTAAAAGATGCTGAAGATCAGTTGGGTGCACGAGTGGGTTACATCGAACTGGATCTCAACAGCGGTAAGATCCTTGAGAGTTTTCGCCCCGAAGAACGTTTTCCAATGATGAGCACTTTTAAAGTTCTGCTATGTGGTGCGGTATTATCCCGTGTTGACGCCGGGCAAGAGCAACTCGGTCGCCGCATACACTATTCTCAGAATGACTTGGTTGAGTACTCACCAGTCACAGAAAAGCATCTTACGGATGGCATGACAGTAAGAGAATTATGCAGTGCTGCCATAACCATGAGTGATAACACTGCTGCCAACTTACTTCTGACAACGATCGGAGGACCGAAGGAGCTAACCGCTTTTTTGCACAACATGGGGGATCATGTAACTCGCCTTGATCGTTGGGAACCGGAGCTGAATGAAGCCATACCAAACGACGAGCGTGACACCACGATGCCTGCAGCAATGGCAACAACGTTGCGCAAACTATTAACTGGCGAACTACTTACTCTAGCTTCCCGGCAACAATTAATAGACTGGATGGAGGCGGATAAAGTTGCAGGACCACTTCTGCGCTCGGCCCTTCCGGCTGGCTGGTTTATTGCTGATAAATCTGGAGCCGGTGAGCGTGGGTCTCGCGGTATCATTGCAGCACTGGGGCCAGATGGTAAGCCCTCCCGTATCGTAGTTATCTACACGACGGGGAGTCAGGCAACTATGGATGAACGAAATAGACAGATCGCTGAGATAGGTGCCTCACTGATTAAGCATTGGTAA | AY458016 | 100.00 |
|  | *bla*_SHV-193_ | ATGCGTTATATTCGCCTGTGTATTATCTCCCTGTTAGCCACCCTGCCGCTGGCGGTACACGCCAGCCCGCAGCCGCTTGAGCAAATTAAACAAAGCGAAAGCCAGCTGTCGGGCCGCGTAGGCATGATAGAAATGGATCTGGCCAGCGGCCGCACGCTGACCGCCTGGCGCGCCGATGAACTCTTTCCCATGATGAGCACCTTTAAAGTAGTGCTCTGCGGCGCAGTGCTGGCGCGGGTGGATGCCGGTGACGAACAGCTGGAGCGAAAGATCCACTATCGCCAGCAGGATCTGGTGGACTACTCTCCGGTCAGCGAAAAACACCTTGCCGACGGCATGACGGTCGGCGAACTCTGTGCCGCCGCCATTACCATGAGCGATAACAGCGCCGCCAATCTGCTGCTGGCCACCGTCGGCGGCCCCGCAGGATTGACTGCCTTTTTGCGCCAGATCGGCGACAACGTCACCCGCCTTGACCGCTGGGAAACGGAACTGAATGAGGCGCTTCCCGGCGACGCCCGCGACACCACTACCCCGGCCAGCATGGCCGCGACCCTGCGCAAGCTGCTGACCAGCCAGCGTCTGAGCGCCCGTTCGCAACGGCAGCTGCTGCAGTGGATGGTGGACGATCGGGTCGCCGGACCGTTGATCCGCTCCGTGCTGCCGGCGGGCTGGTTTATCGCCGATAAGACCGGAGCTGGCGAGCGGGGTGCGCGCGGGATTGTCGCCCTGCTTGGCCCGAATAACAAAGCAGAGCGCATTGTGGTGATTTATCTGCGGGATACGCCGGCGAGCATGGCCGAGCGAAATCAGCAAATCGCCGGGATCGGCGCGGCGCTGATCGAGCACTGGCAACGCTAA | KR347170 | 100.00 |
| KP-54-1 | *bla*_CTX-M-15_ | ATGGTTAAAAAATCACTGCGCCAGTTCACGCTGATGGCGACGGCAACCGTCACGCTGTTGTTAGGAAGTGTGCCGCTGTATGCGCAAACGGCGGACGTACAGCAAAAACTTGCCGAATTAGAGCGGCAGTCGGGAGGCAGACTGGGTGTGGCATTGATTAACACAGCAGATAATTCGCAAATACTTTATCGTGCTGATGAGCGCTTTGCGATGTGCAGCACCAGTAAAGTGATGGCCGCGGCCGCGGTGCTGAAGAAAAGTGAAAGCGAACCGAATCTGTTAAATCAGCGAGTTGAGATCAAAAAATCTGACCTTGTTAACTATAATCCGATTGCGGAAAAGCACGTCAATGGGACGATGTCACTGGCTGAGCTTAGCGCGGCCGCGCTACAGTACAGCGATAACGTGGCGATGAATAAGCTGATTGCTCACGTTGGCGGCCCGGCTAGCGTCACCGCGTTCGCCCGACAGCTGGGAGACGAAACGTTCCGTCTCGACCGTACCGAGCCGACGTTAAACACCGCCATTCCGGGCGATCCGCGTGATACCACTTCACCTCGGGCAATGGCGCAAACTCTGCGGAATCTGACGCTGGGTAAAGCATTGGGCGACAGCCAACGGGCGCAGCTGGTGACATGGATGAAAGGCAATACCACCGGTGCAGCGAGCATTCAGGCTGGACTGCCTGCTTCCTGGGTTGTGGGGGATAAAACCGGCAGCGGTGGCTATGGCACCACCAACGATATCGCGGTGATCTGGCCAAAAGATCGTGCGCCGCTGATTCTGGTCACTTACTTCACCCAGCCTCAACCTAAGGCAGAAAGCCGTCGCGATGTATTAGCGTCGGCGGCTAAAATCGTCACCGACGGTTTGTAA | AY044436 | 100.00 |
|  | *bla*_TEM-1_ | ATGAGTATTCAACATTTTCGTGTCGCCCTTATTCCCTTTTTTGCGGCATTTTGCCTTCCTGTTTTTGCTCACCCAGAAACGCTGGTGAAAGTAAAAGATGCTGAAGATCAGTTGGGTGCACGAGTGGGTTACATCGAACTGGATCTCAACAGCGGTAAGATCCTTGAGAGTTTTCGCCCCGAAGAACGTTTTCCAATGATGAGCACTTTTAAAGTTCTGCTATGTGGTGCGGTATTATCCCGTGTTGACGCCGGGCAAGAGCAACTCGGTCGCCGCATACACTATTCTCAGAATGACTTGGTTGAGTACTCACCAGTCACAGAAAAGCATCTTACGGATGGCATGACAGTAAGAGAATTATGCAGTGCTGCCATAACCATGAGTGATAACACTGCTGCCAACTTACTTCTGACAACGATCGGAGGACCGAAGGAGCTAACCGCTTTTTTGCACAACATGGGGGATCATGTAACTCGCCTTGATCGTTGGGAACCGGAGCTGAATGAAGCCATACCAAACGACGAGCGTGACACCACGATGCCTGCAGCAATGGCAACAACGTTGCGCAAACTATTAACTGGCGAACTACTTACTCTAGCTTCCCGGCAACAATTAATAGACTGGATGGAGGCGGATAAAGTTGCAGGACCACTTCTGCGCTCGGCCCTTCCGGCTGGCTGGTTTATTGCTGATAAATCTGGAGCCGGTGAGCGTGGGTCTCGCGGTATCATTGCAGCACTGGGGCCAGATGGTAAGCCCTCCCGTATCGTAGTTATCTACACGACGGGGAGTCAGGCAACTATGGATGAACGAAATAGACAGATCGCTGAGATAGGTGCCTCACTGATTAAGCATTGGTAA | AY458016 | 100.00 |
|  | *bla*_SHV-28_ | ATGCGTTATATTCGCCTGTGTATTATCTCCCTGTTAGCCACCCTGCCGCTGGCGGTACACGCCAGCCCGCAGCCGCTTGAGCAAATTAAACTAAGCGAAAGCCAGCTGTCGGGCCGCGTAGGCATGATAGAAATGGATCTGGCCAGCGGCCGCACGCTGACCGCCTGGCGCGCCGATGAACGCTTTCCCATGATGAGCACCTTTAAAGTAGTGCTCTGCGGCGCAGTGCTGGCGCGGGTGGATGCCGGTGACGAACAGCTGGAGCGAAAGATCCACTATCGCCAGCAGGATCTGGTGGACTACTCGCCGGTCAGCGAAAAACATCTTGCCGACGGCATGACGGTCGGCGAACTCTGCGCCGCCGCCATTACCATGAGCGATAACAGCGCCGCCAATCTGCTGCTGGCCACCGTCGGCGGCCCCGCAGGATTGACTGCCTTTTTGCGCCAGATCGGCGACAACGTCACCCGCCTTGACCGCTGGGAAACGGAACTGAATGAGGCGCTTCCCGGCGACGCCCGCGACACCACTACCCCGGCCAGCATGGCCGCGACCCTGCGCAAGCTGCTGACCAGCCAGCGTCTGAGCGCCCGTTCGCAACGGCAGCTGCTGCAGTGGATGGTGGACGATCGAGTCGCCGGACCGTTGATCCGCTCCGTGCTGCCGGCGGGCTGGTTTATCGCCGATAAGACCGGAGCTGGCGAACGGGGTGCGCGCGGGATTGTCGCCCTGCTTGGCCCGAATAACAAAGCAGAGCGCATCGTGGTGATTTATCTGCGGGATACCCCGGCGAGCATGGCCGAGCGAAATCAGCAAATCGCCGGGATCGGCGCGGCGCTGATCGAGCACTGGCAACGCTAA | AF299299 | 99.76 |
| KP-PD42 | *bla*_TEM-1_ | ATGAGTATTCAACATTTTCGTGTCGCCCTTATTCCCTTTTTTGCGGCATTTTGCCTTCCTGTTTTTGCTCACCCAGAAACGCTGGTGAAAGTAAAAGATGCTGAAGATCAGTTGGGTGCACGAGTGGGTTACATCGAACTGGATCTCAACAGCGGTAAGATCCTTGAGAGTTTTCGCCCCGAAGAACGTTTTCCAATGATGAGCACTTTTAAAGTTCTGCTATGTGGTGCGGTATTATCCCGTGTTGACGCCGGGCAAGAGCAACTCGGTCGCCGCATACACTATTCTCAGAATGACTTGGTTGAGTACTCACCAGTCACAGAAAAGCATCTTACGGATGGCATGACAGTAAGAGAATTATGCAGTGCTGCCATAACCATGAGTGATAACACTGCTGCCAACTTACTTCTGACAACGATCGGAGGACCGAAGGAGCTAACCGCTTTTTTGCACAACATGGGGGATCATGTAACTCGCCTTGATCGTTGGGAACCGGAGCTGAATGAAGCCATACCAAACGACGAGCGTGACACCACGATGCCTGCAGCAATGGCAACAACGTTGCGCAAACTATTAACTGGCGAACTACTTACTCTAGCTTCCCGGCAACAATTAATAGACTGGATGGAGGCGGATAAAGTTGCAGGACCACTTCTGCGCTCGGCCCTTCCGGCTGGCTGGTTTATTGCTGATAAATCTGGAGCCGGTGAGCGTGGGTCTCGCGGTATCATTGCAGCACTGGGGCCAGATGGTAAGCCCTCCCGTATCGTAGTTATCTACACGACGGGGAGTCAGGCAACTATGGATGAACGAAATAGACAGATCGCTGAGATAGGTGCCTCACTGATTAAGCATTGGTAA | AY458016 | 100.00 |
|  | *bla*_SHV-27_ | ATGCGTTATATTCGCCTGTGTATTATCTCCCTGTTAGCCACCCTGCCGCTGGCGGTACACGCCAGCCCGCAGCCGCTTGAGCAAATTAAACTAAGCGAAAGCCAGCTGTCGGGCCGCGTAGGCATGATAGAAATGGATCTGGCCAGCGGCCGCACGCTGACCGCCTGGCGCGCCGATGAACGCTTTCCCATGATGAGCACCTTTAAAGTAGTGCTCTGCGGCGCAGTGCTGGCGCGGGTGGATGCCGGTGACGAACAGCTGGAGCGAAAGATCCACTATCGCCAGCAGGATCTGGTGGACTACTCGCCGGTCAGCGAAAAACATCTTGCCGACGGCATGACGGTCGGCGAACTCTGTGCCGCCGCCATTACCATGAGCGATAACAGCGCCGCCAATCTGCTGCTGGCCACCGTCGGCGGCCCCGCAGGATTGACTGCCTTTTTGCGCCAGATCGACGACAACGTCACCCGCCTTGACCGCTGGGAAACGGAACTGAATGAGGCGCTTCCCGGCGACGCCCGCGACACCACTACCCCGGCCAGCATGGCCGCGACCCTGCGCAAGCTGCTGACCAGCCAGCGTCTGAGCGCCCGTTCGCAACGGCAGCTGCTGCAGTGGATGGTGGACGATCGGGTCGCCGGACCGTTGATCCGCTCCGTGCTGCCGGCGGGCTGGTTTATCGCCGATAAGACCGGAGCTGGCGAGCGGGGTGCGCGCGGGATTGTCGCCCTGCTTGGCCCGAATAACAAAGCAGAGCGCATTGTGGTGATTTATCTGCGGGATACGCCGGCGAGCATGGCCGAGCGAAATCAGCAAATCGCCGGGATCGGCGCGGCGCTGATCGAGCACTGGCAACGCTAA | AF293345 | 100.00 |
|  | *bla*_DHA-1_ | ATGAAAAAATCGTTATCTGCAACACTGATTTCCGCTCTGCTGGCGTTTTCCGCCCCGGGGTTTTCTGCCGCTGATAATGTCGCGGCGGTGGTGGACAGCACCATTAAACCGCTGATGGCACAGCAGGATATTCCCGGGATGGCGGTTGCCGTCTCCGTAAAGGGTAAGCCCTATTATTTCAATTATGGTTTTGCCGATATTCAGGCAAAACAGCCGGTCACTGAAAATACACTATTTGAGCTCGGATCTGTAAGTAAAACTTTCACAGGTGTGCTGGGTGCGGTTTCTGTGGCGAAAAAAGAGATGGCGCTGAATGATCCGGCGGCAAAATACCAGCCGGAGCTGGCTCTGCCGCAGTGGAAGGGGATCACATTGCTGGATCTGGCTACCTATACCGCAGGCGGACTGCCGTTACAGGTGCCGGATGCGGTAAAAAGCCGTGCGGATCTGCTGAATTTCTATCAGCAGTGGCAGCCGTCCCGGAAACCGGGCGATATGCGTCTGTATGCAAACAGCAGTATCGGCCTGTTTGGTGCTCTGACCGCAAACGCGGCGGGGATGCCGTATGAGCAGTTGCTGACTGCACGGATCCTGGCACCGCTGGGGTTATCTCACACCTTTATTACTGTGCCGGAAAGTGCGCAAAGCCAGTATGCGTACGGTTATAAAAACAAAAAACCGGTCCGCGTGTCGCCGGGACAGCTTGATGCGGAATCTTACGGCGTGAAATCCGCCTCAAAAGATATGCTGCGCTGGGCGGAAATGAATATGGAGCCGTCACGGGCCGGTAATGCGGATCTGGAAATGGCAATGTATCTCGCCCAGACCCGCTACTATAAAACCGCCGCGATTAACCAGGGGCTGGGCTGGGAAATGTATGACTGGCCGCAGCAGAAAGATATGATCATTAACGGTGTGACCAACGAGGTCGCATTGCAGCCGCATCCGGTAACAGACAACCAGGTTCAGCCGTATAACCGTGCTTCCTGGGTGCATAAAACGGGCGCAACAACTGGTTTCGGCGCCTATGTCGCCTTTATTCCGGAAAAACAGGTGGCGATTGTGATTCTGGCGAATAAAAACTACCCGAATACCGAAAGAGTCAAAGCTGCACAGGCTATTTTGAGTGCACTGGAATAA | Y16410 | 100.00 |
