# Supplementary material for: Prevalence of antimicrobial resistance and virulence genes in Klebsiella pneumoniae and Congenetic Raoultella Isolates from captive giant pandas
Source: PLoS One. 2023 Mar 30;18(3):e0283738. doi: 10.1371/journal.pone.0283738 (PMC10062605; doi:10.1371/journal.pone.0283738)
Supplement: S2 Table — (DOCX) [file pone.0283738.s003.docx]

S2 Table. The sequences of the virulence-related genes.

| **virulence-related gene** | **Isolate** | **Sequence** | **Reference sequence**  **(accession number)** | **Homology**  **(%)** |
| --- | --- | --- | --- | --- |
| *ybtS* | 29 | ATGAAAATCAGTGAATTTCTACATCTGGCGTTACCAGAGGAACAATGGCTACCGACGATTTCTGGCGTTTTACGCCAGTTCGCAGAAGAAGAGTGTTATGTCTATGAGCGTCAACCCTGTTGGTATTTAGGCAAAGGGTGCCAGGCACGGCTGCACATTAATGCCGACGGAACGCAGGCGACATTTATTGATGATGCCGGGGAGCAAAAATGGGCGGTGGATTCCATTTCCGACTGCGCGCGTCGTTTTATGGCGCATCCTCAGGTGAAAGGACGTCGGGTATATGGACAGGTTGGGTTCAACTTTGCGGCGCATGCGCGGGGGATTGCCTTTAACGCCGGGGAGTGGCCGCTGCTGACGTTAACCGTTCCCCGTGAAGAACTTATTTTTGAAAAGGGAAATGTCACCGTTTATGCGGACTCCGCCGACGGGTGCCGACGTTTGTGCGAGTGGGTAAAAGAGGCCGGTACAACGACGCAGAACGCACCACTGGCGGTGGATACCGCCCTCAATGGTGAGGCATATAAACAACAGGTTGCGCGCGCCGTTGCGGAGATCCGCCGTGGCGAGTATGTCAAAGTGATTGTCTCGCGCGCCATTCCCCTGCCATCGCGGATTGATATGCCCGCCACGCTGTTATACGGGCGGCAGGCAAACACACCTGTGCGCTCGTTTATGTTCCGTCAGGAAGGACGCGAAGCGCTGGGCTTTAGCCCGGAACTGGTGATGTCAGTGACGGGCAATAAAGTGGTCACTGAACCGCTTGCGGGCACCCGCGATCGCATGGGAAACCCGGAGCATAATAAGGCGAAAGAGGCAGAACTGCTGCACGACAGTAAAGAGGTGCTTGAGCATATCCTTTCTGTCAAAGAAGCTATTGCTGAACTGGAGGCCGTTTGCCAGCCGGGCAGCGTGGTGGTTGAAGATTTAATGTCGGTTCGCCAGCGCGGCAGCGTTCAGCATCTGGGGTCTGGCGTGAGCGGTCAGCTTGCGGAAAACAAGGATGCCTGGGATGCGTTTACCGTGCTGTTTCCGTCGATTACCGCCTCAGGTATCCCTAAAAATGCTGCTCTGAACGCGATTATGCAAATTGAGAAGACGCCGCGAGAGCTTTATTCCGGCGCAATCCTGCTGCTGGACGATACGCGCTTTGATGCGGCGCTAGTTCTGCGTTCCGTATTTCAGGATAGCCAGCGCTGCTGGATACAGGCGGGGGCGGGAATCATCGCGCAATCTACGCCGGAACGCGAACTGACAGAAACCCGGGAGAAATTAGCGAGCATTGCGCCCTATTTAATGGTGTAG | CP047633 | 100.00 |
|  | 41 | ATGAAAATCAGTGAATTTCTACATCTGGCGTTACCAGAGGAACAATGGCTACCGACGATTTCTGGCGTTTTACGCCAGTTCGCAGAAGAAGAGTGTTATGTCTATGAGCGTCAACCCTGTTGGTATTTAGGCAAAGGGTGCCAGGCACGGCTGCACATTAATGCCGACGGAACGCAGGCGACATTTATTGATGATGCCGGGGAGCAAAAATGGGCGGTGGATTCCATTGCCGACTGCGCGCGTCGTTTTATGGCGCATCCTCAGGTGAAAGGACGTCGGGTATATGGACAGGTTGGGTTCAACTTTGCGGCGCATGCGCGGGGGATTGCCTTTAACGCCGGGGAGTGGCCGCTGCTGACGTTAACCGTTCCCCGTGAAGAACTTATTTTTGAAAAGGGAAATGTCACCGTTTATGCGGACTCCGCCGACGGGTGCCGACGTTTGTGCGAGTGGGTAAAAGAGGCCGGTACAACGACGCAGAACGCACCACTGGCGGTGGATACCGCCCTCAATGGTGAGGCATATAAACAACAGGTTGCGCGCGCCGTTGCGGAGATCCGCCGTGGCGAGTATGTCAAAGTGATTGTCTCGCGCGCCATTCCCCTGCCATCGCAGATTGATATGCCCGCCACGCTGTTATACGGGCGGCAGGCAAACACACCTGTGCGCTCGTTTATGTTCCGTCAGGAAGGACGCGAAGCGCTGGGCTTTAGCCCGGAACTGGTGATGTCAGTGACGGGCAATAAAGTGGTCACTGAACCGCTTGCGGGCACCCGCGATCGCATGGGAAACCCGGAGCATAATAAGGCGAAAGAGGCAGAACTGTTGCACGACAGTAAAGAGGTGCTTGAGCATATCCTTTCTGTCAAAGAAGCTATTGCTGAACTGGAGGCCGTTTGCCAGCCGGGCAGCGTGGTGGTTGAAGATTTAATGTCGGTTCGCCAGCGCGGCAGCGTTCAGCATCTGGGGTCTGGCGTGAGCGGTCAGCTTGCGGAAAACAAGGATGCCTGGGATGCGTTTACCGTGCTGTTTCCGTCGATTACCGCCTCAGGTATCCCTAAAAATGCTGCTCTGAACGCGATTATGCAAATTGAGAAGACGCCGCGAGAGCTTTATTCCGGCGCAATCCTGCTGCTGGACGATACGCGCTTTGATGCGGCGCTAGTTCTGCGTTCCGTATTTCAGGATAGCCAGCGCTGCTGGATACAGGCGGGGGCGGGAATCATCGCGCAATCTACGCCGGAACGCGAACTGACAGAAACCCGGGAGAAATTAGCGAGCATTGCGCCCTATTTAATGGTGTAG | KY454627 | 100.00 |
|  | KP-43 | ATGAAAATCAGTGAATTTTTACACCTGGCGCTACCAGAGGAACAATGGCTGCCGACGATTTCTGGCGTCTTACGCCAGTTCGCAGAAGAAGAGTGCTATGTCTACGAGCGTCAACCCTGTTGGTATTTAGGTAAAGGATGCCTGGCACGGTTGCACATTAATGCCAACGGAACGCAGGCGACATTCATTGATGATGCCGGGGAGCAAAAATGGGCGGTGGATTCCATTGTCGACTGCGCGCGTCGTTTTATGGCGCATCCGCAGGTGCAAGGGCGTCGGGTATATGGGCAGGTTGGGTTCAACTTTGCGGCGCATGTGCGGGGGATTGCCTTCGACGCCGGCGAGTGGCCGCTGCTGACGTTAACCGTTCCCCGTGAAGAACTTATTTTTGAAAAGGGAAATGTCACCGTTTATGCGGACTCCGCTGACGGGTGCCGACGTCTGTGCGAGTGGGTGAAAGAGGCTGGTACAACGACGCAGAACGCACCACTGGTGGTGGATACCGCCCTCAATGGTGAGGCGTATAAACAACAAGTTGCCCGCGCCGTTGCGGAGATACGCCGTGGCGAGTATGTCAAAGTGATTGTCTCGCGCGCCATTCCCCTGCCATCACGGATTGATATGCCCGCCACGCTGTTATACGGGCGGCAGGCAAACACGCCCACGCGGTCGTTTATGTTCCGCCAGCAAGGGCGCGAGGCGCTGGGCTTTAGCCCGGAACTGGTGATGTCAGTGACGGGCAATAAAGTGGTCACTGAACCGCTTGCGGGCACGCGCGATCGCATGGGAAGCCCGGAGCAAAATAAGGCGAAAGAGACAGAGCTGCTGCACGACAGTAAAGAGGTGCTTGAGCATATCCTTTCTGTCAAAGAAGCTCTTGCTGAACTGGCGGTGGTTTGCCGGCCGGGCAGCGTGGTGGTTGAAGATTTAATGTCGGTCCGCCAGCGTGGCAGCGTTCAGCATCTGGGGTCTGGCGTGAGCGGTCAGCTTGCGGAAAACAAAGATGCCTGGGATGCATTTACCGTGCTGTTCCCGTCGATTACCGCCTCGGGTATCCCTAAAAATGCTGCCCTGAACGCCATTATGCGAATTGAGAAGACCCCGCGAGAGCTCTATTCCGGCGCAATCCTGCTACTGGAAGATGCGCGCTTCGATGCGGCGTTAGTCCTGCGTTCCGTATTTCAGGACAGTCAACGGTGCTGGATACAGGCGGGAGCAGGGATCATTGCCCAATCTACGCCGGAACGTGAACTGACGGAAACCCGGGAGAAATTAGCGAGCATTGCGCCCTATCTCATGGTATCGGAGTGA | CP104450 | 96.34% |
|  | KP-PD111 | ATGAAAATCAGTGAATTTTTACACCTGGCGCTACCAGAGGAACAATGGCTGCCGACGATTTCTGGCGTCTTACGCCAGTTCGGAGATGAAGAGTGCTATGTCTACGAGCGTCAACCCTGTTGGTATTTAGGTAAAGGATGCCTGGCACGGTTGCACATTAATGCCGACGGAACGCAGGCGACATTCATTGATGATGCCGGGGAGCAAAAATGGGCGGTGGATTCCATTGTCGACTGCGCGCGTCGTTTTATGGCGCATCCGCAGGTGCAAGGGCGTCGGGTATATGGGCAGGTTGGGTTCAACTTTGCGGCGCATGCGCGGGGGATTGCCTTCGACGCCGGCGAGTGGCCGCTGCTGACATTAACCGTTCCCCGGGAAGAGCTTGTGTTTGAAAAGGGAAATGTCACCGTTTATACGGACTCCGCTGAGGGATGTCGACGTCTGTGCGAGTGGGTAAAAGAGGCCAGTACAACCACGCGAGGGGACTCCATGGCTGTGGAAACCGCCCTCAATGGTGAGGTGTATAAACAACAGGTGGCGCGCGCCGTTGCGGAGATCAGCCGGGGCGAATATGTCAAAGTGATTATCTCGCGCGCCATTCCGCTGCCATCGCGGATTGATATGCCCGCCACCCTGTTATACGGGCGGCAGGCAAACACGCCCACGCGGTCGTTTATGTTCCGCCAGCAAGGACGCGAAGCGCTAGGGTTTAGCCCGGAGCTGGTGATGTCGGTAACGGGCAATAAAGTGGTCACTGAACCGCTTGCGGGCACCCGCGATCGCATGGGAAGCCCGGAGCAAAATAAGGCGAAAGAGACAGAGCTGCTGCACGACAGTAAAGAGGTGCTTGAGCATATCCTTTCTGTCAAAGAAGCGCTTGCTGAACTGGCGGTGGTTTGCCGGCCGGGCAGCGTGGTGGTTGAAGATTTAATGTCGGTCCGCAAGCGTGGCAGCGTTCAGCATCTGGGGTCTGGCGTGAGCGGTCAGCTTGCGGAAAACAAAGATGCCTGGGATGCATTTACCGTGCTGTTCCCGTCGATTACCGCCTCGGGTATCCCTAAAAATGCTGCTCTGAACGCCATTATGCGAATTGAGAAGACCCCGCGAGAGCTCTATTCCGGCGCAATCCTGCTACTGGAAGATGCGCGCTTCGATGCGGCGTTAGTCCTGCGTTCCGTATTTCAGGACAGTCAACGGTGCTGGATACAGGCGGGAGCAGGGATCATTGCCCAATCTACGCCGGAACGTGAACTGACGGAAACCCGGGAGAAATTAGCGAGCATTGCGCCCTATCTCATGGTATCGGAGTGA | CP080266 | 99.77% |
|  | KP-22 | ATGAAAATCAGTGAATTTCTACATCTGGCGTTACCAGAGGAACAATGGCTACCGACGATTTCTGGCGTTTTACGCCAGTTCGCAGAAGAAGAGTGTTATGTCTATGAGCGTCAACCCTGTTGGTATTTAGGCAAAGGGTGCCAGGCACGGCTGCACATTAATGCCGACGGAACGCAGGCGACATTTATTGATGATGCCGGGGAGCAAAAATGGGCGGTGGATTCCATTGCCGACTGCGCGCGTCGTTTTATGGCGCATCCTCAGGTGAAAGGACGTCGGGTATATGGACAGGTTGGGTTCAACTTTGCGGCGCATGCGCGGGGGATTGCCTTTAACGCCGGGGAGTGGCCGCTGCTGACGTTAACCATTCCCCGTGAAGAACTTATTTTTGAAAAGGGAAATGTCACCGTTTATGCGGACTCCGCCGACGGGTGCCGACGTTTGTGCGAGTGGGTAAAAGAGGCCGGTACATCGACGCAGAACGCACCACTGGCGGTGGATACCGCCCTCAATGGTGAGGCATATAAACAACAGGTTGCGCGCGCCGTTGCGGAGATCCGCCGTGGCGAGTATGTCAAAGTGATTGTCTCGCGCGCCATTCCCCTGCCATCGCGGATTGATATGCCCGCCACGCTGTTATACGGGCGGCAGGCAAACACACCTGTGCGCTCGTTTATGTTCCGTCAGGAAGGACGCGAAGCGCTGGGCTTTAGCCCGGAACTGGTGATGTCAGTGACGGGCAATAAAGTGGTCACTGAACCGCTTGCGGGCACCCGCGATCGCATGGGAAACCCGGAGCATAATAAGGCGAAAGAGGCAGAACTGCTGCACGACAGTAAAGAGGTGCTTGAGCATATCCTTTCTGTCAAAGAAGCTATTGCTGAACTGGAGGCCGTTTGCCAGCCGGGCAGCGTGGTGGTTGAAGATTTAATGTCGGTTCGCCAGCGCGGCAGCGTTCAGCATCTGGGGTCTGGCGTGAGCGGTCAGCTTGCGGAAAACAAGGATGCCTGGGATGCGTTTACCGTGCTGTTTCCGTCGATTACCGCATCAGGTATCCCTAAAAATGCTGCTCTGAACGCGATTATGCAAATTGAGAAGACGCCGCGAGAGCTTTACTCCGGCGCAATCCTGCTGCTGGACGATACGCGCTTTGATGCGGCGCTAGTTCTGCGTTCCGTATTTCAGGATAGCCAGCGCTGCTGGATACAGGCGGGGGCGGGAATCATCGCGCAATCTACGCCGGAACGCGAACTGACAGAAACCCGGGAGAAATTAGCGAGCATTGCGCCCTATTTAAAGGTGTAG | CP110748 | 99.92% |
| *iutA* | 29 | ATGGATATGAAAAAGCGCCTCTGGGTGCTCCACCCCCTGCTGCTGGTCAGCTCGCTGCCTGCGCTGGCGGCTCAGTCTGATGAAGACAGCATCATCGTTAGTGCAAACCGCACCCATCGCACCGTGGCCGAAATGGCCCAAACCACCTGGGTCATTGAGGGCCAGGAGATTGAGCAGCAGGTCCAGGGCGGGAAAGAGTTCAAAGACGTGCTGGCGCAGCTGATCCCGGGCATCGATGTCAGCAGCCAGGGGCGCACCAATTACGGGATGAACATGCGCGGGCGCGCCATCGTCGTGCTGATCGACGGCGTCCGGCTCAATTCCTCACGCACCGACAGTCGCCAGCTCGACGCCATCGACCCCTTTAACATCGAACATATCGAAGTGATCTCCGGGGCGACCTCGCTGTACGGCGGAGGCAGTACCGGCGGGCTTATCAACATCGTCACCAAGAAAGGGCAGCAGGATCGCCAGGTCGATCTCGAAGTGGGCAGCAAGAGCGGGTTTGCGAACAGTAACGATCACGATGAGCGCATCGCGGCGGCCGTCAGCGGCGGGACAGACCATGCATCCGGGCGCTTATCGGTAGCCTATCAGCGTTTCGGCGGCTGGTACGATGGCAATAACGATGCGCTGATCCTCGATAACACGCAAACGGGGCTCCAGCACTCTGACCGCCTCGACGTGATGGGGACGGGGACGATTGAGATCGATGACAATCGCCAGTTGCAGCTTGTCACCCAGTATTATAAAAGCCAGGGCGATGATGACTACGGCCTGTGGCTCGGGAAGAACATGTCCGCGGTCACCAGCGGCGGCAAAGCGTACACCACCGACGGGCTCAATTCCGACCGTATCCCCGGTACAGAGCGGCACTTAATCAGCCTCCAGTACTCCGATGCCGACTTCTTCGGCCAGAATCTGGTGAGCCAGGTGTACTATCGCGATGAGTCCCTCACCTTCTATCCGTTCCCGACGCTTACGAAAGGCCAGGTCAGTAGCTTCTCCTCGTCGCAGCAGGATACCGATCAGTATGGGGCAAAGCTGACCCTTAACAGCCAGCCGCTGGCGGGATGGGATCTCACCTGGGGTCTCGACGCCGATCATGAGACCTTCAATGCCAACCAGATGTTCTTCGATCTGCAGCAATCGCTGGCGTCCGGCGGGCTGCACAACGAATCGATCTACACCACCGGCCGCTATCCGGGATACAGCATCTCCAACGTCGCGCCGTTCCTGCAGTCCAGCTACGATCTGAACGAGATCTTTACCGTCAGCGGCGGGGTACGCTACCAGTGGACAGAAAACCGGGTTGACGACTTTGTCGGCTACGCCCAGCAACAGGATATCGCCAACGGCAAAGCGCGCTCCGCCGACGCCATCAAAGGCGGCAAAACCGATTACGATAACTTCCTGTTTAACGCCGGGATCGTGGCGCATCTGACCGAGCGTCAGCAGACCTGGTTTAACTTCTCGCAGGGCGTCGAGCTGCCGGACCCCGGTAAATACTACGGCATCGGTAAATACGGCGCGGCGGTCAATGGCCATCTGCCGCTGATCTCCAGCGTCAACGTCGATGACTCGCCGCTGCAGGGGATCAAAGTTAACTCGTATGAGCTGGGCTGGCGCTACACCGGCGATAACCTGCGCACCCAGCTGGCGGCATATTACTCGACTTCGGATAAGACCATCGTCGTCAACCGCACCGACATGACCATCGACGTGCAGTCCGACAAACGGCGTATTTACGGCGTTGAGGGAGCGGTCGATTACTTTATTCCGGACAGCGACTGGAGCGTCGGCGGTAACTTCAACGTGCTGAAATCACAGGTGCAGACCGACGGCCGCTGGCAAAAATGGGATGTCACCCTCGCCTCACCGTCCAAAGCCACCGCCTGGGTGGGTTGGGCGCCGGATCCGTGGAGCCTGCGCGTGCAGAGCCAGCAGGTGTTTGACCTCAGCGACGCCGCCGGTAACAAGCTGGAAGGCTATAACACCGTTGATTTTATTGGCAGTTACGCGCTGCCGGTGGGGAAACTGACCTTCAGTATCGAAAACCTGCTTAACGAAGACTATGTCACCATCTGGGGCCAGCGGGCGCCGCTGCTCTACAGCCCGACCTACGGCAGTTCATCGCTGTACGAATACAAAGGCCGTGGCCGTACCTTCGGTCTGAATTACGCGTTAACCTTCTGA | LR134235 | 99.86 |
|  | KP-41-1 | ATGGATATGAAAAAGCGCCTCTGGGTGCTCCACCCTCTGCTGCTGGCCAGCGCGCTGCCTGCGCTGGCGGCTCAGTCTGAAGAAGACAGCATCATCGTTAGCGCGAACCGCACCCATCGCACCGTAGCCGAAATGGCCCAAACCACCTGGGTCATTGAGGGCCAGGAAATTGAGCAGCAGGTCCAGGGCGGAAAAGAGTTCAAAGACGTGCTGGCGCAGCTGATCCCGGGCATCGACGTCAGCAGCCAGGGGCGCACCAACTATGGGATGAACATGCGCGGGCGCGCCATCGTCGTGCTGATTGACGGCGTCCGGCTCAACTCTTCCCGCACCGACAGCCGCCAGCTCGACGCCATCGACCCGTTCAACATCGAGCATGTCGAAGTGATCTCCGGGGCAACCTCGCTGTACGGCGGCGGCAGTACCGGCGGACTTATCAACATCGTCACCAAAAAAGGGCAGCAGGATCGCCAGGTCGATCTTGAGGTGGGCAGCAAGAGCGGGTTTGCAAACAGCAACGATCATGATGAGCGTGTCGCGGCGGCGGTCAGCGGCGGGACAGACCATGCGTCCGGGCGTTTATCGGTAGCCTATCAGCGTTTCGGCGGTTGGTACGACGGCAATAACGATGCGCTGATCCTCGATAACACCCAAACGGGGCTCCAGCACTCTGATCGTCTGGACGTCATGGGGACGGGGACGATCGAGATCGACGACAACCGCCAGCTGCAGCTGGTCACCCAGTACTATAAAAGCCAGGGCGATGATGACTATGGCCTGTGGCTCGGGAAAAATATGTCCGCGGTCACCAGCGGCGGCAAAGCGTACACCACCGATGGGCTCAATTCCGATCGTATCCCCGGCACCGAGCGCCATCTGATCAGCCTCCAGTACTCCGATGCCGATTTCTTCGGCCAGAATCTGGTGAGCCAGGTGTACTATCGCGATGAGTCCCTCACCTTCTATCCATTCCCGACGCTCAGCAAAGGTCAGGTCAGCAGCTTCTCCTCGTCGCAGCAGGATACCGATCAGTACGGGGCAAAGCTGACCCTCAACAGCCAGCCGCTGGCGGGATGGGATCTCACCTGGGGTCTCGACGCCGATCATGAGACCTTCAATGCCAACCAGATGTTCTTCGATCTGCAACAATCGCTGGCGTCCGGCGGGCTGCACAACGAATCGATCTACACCACCGGTCGCTATCCGGGATACAGTATCTCCAACGTCGCGCCGTTCCTGCAGTCGAGCTATGATCTGAACGATATCTTTACCCTCAGCGGCGGGGTTCGCTACCAGTGGACCGAAAACCGGGTTGACGACTTTGTCGGCTACGCCCAGCAACAGGATATCGCGAACGGCAAAGCGCGCTCCGCCGACGCCATCAAAGGCGGCAAAACCGATTATGACAACTTCCTGTTTAACGCCGGGATCGTGGCCCATCTGACCGAGCGTCAGCAGACCTGGTTTAACTTCTCGCAGGGCGTTGAGCTGCCGGATCCGGGTAAATACTATGGCATCGGGAAATACGGCGCGGCGGTCAATGGTCATCTGCCGCTGCTCTCCAGCGTCAACGTCGATGACTCGCCGCTGCAGGGGATCAAGGTTAACTCGTATGAGCTGGGCTGGCGCTATACCGGCGATAACCTGCGCACTCAGCTGGCGGCGTATTACTCGACTTCGGATAAGACCATCGTCGTCAACCGTACCGATATGACCATCGATGTTCAGTCCGACAAACGGCGTATTTACGGCGTTGAGGGTGCGGTCGATTACTTTATTCCGGACAGCGACTGGAGCGTCGGCGGCAACTTCAACGTGCTGAAATCACAGGTGCAGACCGATGGCCGCTGGCAGAAGTGGGACGTCACCCTCGCCTCGCCGTCCAAAGCCACCGCCTGGGTGGGCTGGGCGCCGGATCCGTGGAGCCTGCGCGTGCAGAGCCAGCAGGTGTTTGACCTCAGCGACGCCGCCGGCAACAAGCTGGAAGGCTATAACACCGTCGATTTTATCGGCAGTTACGCGCTGCCGGTGGGAAAACTGACCTTCAGTATCGAAAACCTGCTTAACGAAGACTATATCACCATCTGGGGCCAGCGCGCGCCGCTGCTCTACAGCCCAACCTACGGCAGCTCGTCGCTGTATGAATACAAGGGCCGCGGCCGCACCTTCGGTCTGAATTACGCGTTAACCTTCTGA | LR588411 | 99.73% |
| *rmpA* | 41 | CATTTGTAAGAGTATTATAATCAATAGTTATTAAGCACAAAAAAAACATAAGAGTATTGGTTGACTGCAGGATTTTTTATTCAGGAAAATGGAGAGGGTACAAAATGTTAAGGAAATCATTAAATATGATAAGCCAATGGATATGGCTTGATGTTTCGGGGGGGGGCGGGTTTTACCCTAAAGG | KY403897 | 96.26% |
| *iroN* | 41 | GTGGAAGCCACCGCTGAGCAGCTACTAAAACAGCAGCCGGGCGTGTCGATTATTACCAGCGAAGATATTATAAAGAACCCTCCGGTCAACGACCTCTCCGATATTATTCGCAAAATGCCTGGTGTCAATCTGACCGGCAATAGCGCCTCGGGGACACGCGGTAATAACCGCCAGATCGACATGCGCGGTATGGGGCCGGAAAACACATTAATTTTAATTGATGGTGTACCGGTGACGTCGCGCAACTCCGTGCGTTATAGCTGGCGCGGGGAGCGTGATACCCGCGGCGACACCAACTGGGTGCCACCAGAACAGGTTGAGCGTATCGAAGTGATCCGCGGCCCTGCGGCGGCGCGCTACGGTTCGGGCGCCGCGGGCGGGGTGGTAAACATCATTACCAAACGCCCCTCCAACGACTGGCACGGTTCGCTGTCGCTATACACCAACCAGCCAGAAAGCAGCGATGAAGGCGCTACGCGTCGCGCCAACTTCAGCCTTAGTGGGCCGCTGGCTGGCGATGCGCTGACCATGCGCCTCTACGGTAACCTGAATAAAACCGATGCCGACAGCTGGGATATTAACTCCTCGGCCGGAACGAAAAACGCGGCCGGGCATGAAGGGGTGCGCAACAAAGATATTAACGGCGTTGTTTCGTGGAAATTAAATCCGCAGCAAATTCTTGATTTCGAAGCCGGATATAGTCGTCAGGGGAATATTTATGCGGGCGATACGCAAAACAGTTCTTCTAGTGCCGTAACTGAAAGCTTGGCGAAATCCGGCGAAGAGACGAACCGCCTGTACCGGCAGAATTATGGCATTACGCATAATGGCATCTGGGACTGGGGACAAAGCCGTTTTGGTGTCTATTACGAGAAAACCAATAACACCCGCATGAATGAAGGATTATCCGGCGGCGGTGAAGGGCGTATTTTGGCGGATGAAAAGTTCACAACCAATCGCCTGAGCACCTTGCGAACCAGCGGCGAGCTTAATATTCCGCTGAATACGCTGGTCGATCAAACGCTGACCGTGGGGGCGGAGTGGAGCCGCGATGAACTCGACGATCCTTCCTCTACCAGCCTGACGGTGGATGACAGTGATATCGGCGGCATTTCTGGCTCGGCTGCGGATCGCAGCAGTAAAAACCATTCCCAAATCAGTGCGCTGTATATTGAAGATAATATTGAGCAGGTTCCCGGCACGAATATAATTCCCGGCTTGCGCTTTGATTATCTCAACGAGTCCGGCGGTAACTTCAGCCCCAGCCTTAACCTCTCGCAGGAATTGGGCGATTACTTCAAAGTCAAAGCGGGGATTGCCCGAACCTTCAAAGCACCAAACTTGTATCAATCCAGCGAAGGTTATCTGCTCTACTCGAAAGGCAACGGTTGTCCAAAAGATATTACATCGGGTGGTTGCTACCTGATCGGTAATAAAAATCTCGATCCGGAAATCAGTATCAATAAAGAAATTGGGCTGGAGTTCGCCTGGGAAGATTACCACGCCAGCGTGACCTACTTCCGCAATGACTACCAGAATAAGATCTTGGCCGGGGATAATGTTATCGGGCAAACCGCTTCAGGCACATATATCCTCCAGTGGCAGAATGGAGGAAAAGCGCTGGTGGACGGTATCGAAGCCAGCATGGCGTTCCCGCTGGTGAAAGATCGTCTGAACTGGAATACCAATGCCACCTGGATGATCACCTCGGAGCAAAAAGACACAGGTAACCCGCTGTCGGTCATCCCGAAATATACCATTAATAACTCGCTTGACTGGACCATCACCCAAGCGTTTTCCGCCAGCGTCAACTGGACGTTATACGGCAGACAAAAACCGCGTACCCATGCGGAAACTCGGAGTGAAGATACTGGCGGCCTGTCAGGTAAAGAGCTGGGAGCTTATTCGCTGGTGGGGACGAACTTCAATTACGATATTAATAAAAATCTTCGTCTTAACGTCGGTGTTAGCAATATCTTCGATAAACAGATTTACCGATCTTCCGAAGGGGCGAATACTTATAACGAGCCTGGCCGGGCTTATTATGCCGGTGTCACCGCATCATTCTGA | CP041353 | 100.00 |
| *iroB* | 41 | GTGCTTTCTCTGGCACAGGCGTTTCGCGTTAACGGCCATGAAGTGCTGATCGCCAGCGGAGGAACATTCGTCAAAAAAGCAGCAGAGGCTGGATTGGTGGCGTTTGATGCCGCACCAGGCTTAGATTCCGAAGCTGGCTATTGCCGCCATGAAGCTCTGCGGAAAGAAAGTCACATTGGCACCAAAATGGGCAACTTCTCATTCTTCAGCGAAGAGATGGCCGACCACTTGGTGGAGTTTGCCGGACACTGGCGGCCCGACCTTATCATCTACCCTCCGCTCGGAGTCATCGGGCCGCTGATTGCGGCCAAATACGATATACCGGTCGTGATGCAAACCGTCGGCTTCGGCCATACGCCTTGGCATATCAAAGGCGTAACGCGTTCGCTTGCGGACGCGTATCATCGCCACGGCGTCGGGGCCGCTCCGCGCGATATGGCCTGGCTCGACGTAACACCGCCGAGTATGAGCATTCTTGAAAACGACGGCGAACCCATTATTCCCATGCAGTACGTCCCGTATAACGGCGGCGCGGTGTGGGAACCGTGGTGGGAGAGGAAGCCCGATCGTAAACGTCTGCTGGTGAGTCTAGGCACCGTCAAACCGATGGTCGACGGCCTTGAATTGATCACTTGGGTGATGGATTCCGCCAGTGAAGTCGATGCCGAGATCATCCTGCATCTTTCGGCCAATGCGCGGTCCGAGCTACGCTCACTACCATCAAATGTCCGCCTGGTCGACTGGATACCCATGGGGGCTTTCCTGAACGGCGCAGATGGCTTCATTCATCATGGCGGCGCAGGCAATACTCTGACAGCGCTACACGCCGGTATTCCACAGATCGTCTTCGGCCAGGGTGCTGATCGCCCGGTAAATGCCCGCGTTGTTGTCGAGCGCGGCTGCGGGATCATCCCCGGCGACGGTGGCCTGTCAAGCAACATGATCAATGCTTTCCTCGACAATCGCGCGCTTCGCGACGCCTCTGAAGAGGTAGCGGCGGAAATGGCGGCGCAACCCTGCCCAAGCGAAGTAGCGAAAAAACTGATAGCGATGGTACAGAAAGGGTAG | KY454627 | 100.00 |
| *K2* | KP-54-1 | TTTCTTGTTTTTTATTTTTTGGCGCTTTGCTATTCATTGCGACAAAATATCAAAGATATTCGAGCTGGCGCCGCCTTAATTATTATTGTTGTTATTTTTTCTGTTTTGCTCAGTTATTTAGATCCTGCCATAATAAATAACAAAGGTATTATACAATATAACGATTTTATTAGCCCTGAGAGGTTGAGAGGATTCTCTCTTGAAGCTAGTGTATTTGGCTACCAGATTGTATGTAGTATCCTTCTATTAGCCGTACTGTTAAACTGGAGTACTTTTTTCCTTGTCACTGTAACGATCGTTATCGCTATATTGACAACCTCTAAAGGAGCTGCTCTTTCATTTTTGATATGTATTTGCTTTTATTTTTCCCTTAAAGGAAAGTTAATGTTTAGGGTTTTGCTGTCGCTATGCTCAATAGTAATATCTTATATTATATTCAAATATTATATCCTAGATGCACTTGCAAGTGATATAGACACATATTCATCCGTGGCAACAAGGGGAACGATGTTCATAGTCGGACTGAAGATTTTTTTATTTAATCCGTTAGGAGTTGGATTCTTCGGTTATTTGCCATCTATTTACTTTT | LT174548 | 99.31% |
| *K5* | 41 | CAGAGTTTCGGTAACGAAGAGAAGGGAACACCGCCACCTCTAAGCATATAGCTGAATATATAGAATTTGATAAAAATAAGGTAGGCGCGGGAATCGTGGCAACATTAATTTTTTCTATGTTGTTCGCATCAACTATTTGTCTTTGCGCCAATTTTTTTCCTAATGAATTTTCTATATACTTGCTGTTTCATGAAGACTATTTGATATTTGTTAGCAGTACAGCTGGTATTATTGTTCTTACGATTGGATTGGGGTGGGTTCAGG | AB289646 | 99.58% |
| *K54* | KP-35 | GAGTATAGTTCTATACCGCGCTTGTTAGGCTCAGAAGAAAATGCACAGAAAAGATTAGCGTGCTTTATTAATGCGAGATTTATTTTTGTCACAATTATTGTTATAAGTGTTCTAGTTTATCTTATTACTAAAAATTCGTGGCATCTGTACTACGGACTCGTATCATTTATAATTCCTGTTTTGCTAATTTCATCTTTTAGTGTGATTGGCTATCTCGATTTTTATAATGAAACGAAGGCTTCTGGAACATTGTCTGGAGTGTCTTGGTCATTAGCCTCATTGCCTTTGTATTATTCATATTTCTATGAAGGAGATAGATATATCATAGGTATGAGTGCTGGGCTGCTTTTTGTTTTAGGAAACGCATTATTTATTATAATTCAATGCGTTAAAATGAAACAAGTAAGCAAGAATGGCGGGATTAAATTAATGTATAAGGATGGTTTTTGCATAAAAAAAATAAAATATTCATTTTATGATATCATTAAATATAATTTGAACTATGGGTTTTCACAAAGTTATGGAAGGTTCGTCCCGATTTTATTAAATACACTTTTAGGCCCTGTACTGGCTGGGTATTACATATATGCTAAGAGTTTTGCAAACATTGCAAATCAATTTATAGCTTTCTCCAGAAGAGTTGAGTTTAGTAATCTGATCGCAATTCATAATGGAAGTGTCAGGAAAATATTATCCAGTCAGTTAATTAGCTTTGGAATTGCATGCATATTTGCGATATTTTGTTTTGGAACTGAACTTATCGAGTGTTTTTACACATTGCCTATTGGAAATAATACTCTATTATATATACAAATTATAGCGTTAATTAATCTGCTTGGTG | AB924591 | 99.88% |
| *K57* | KP-28 | CCTGGATTGCATTTTTTTTCTGCTTGTAAAAAAACCAAAGTGTATGATATTTCGTTTTTT8GCAAACCTTTTTTTTACATTATGGGCTGTATGGGTACTTTGTGGTATAACCTACAGCTATTTTACATTCCCGGGGGTTGTTGATCATCAGGCGTCATATCTCAGGTATATTCGGCTGTTGGAAATGTATTTGCCAGGATGTATGATTTTATCTTTCCTTGGTGAGCTATCCAATTCTGTTCATAAAAAGATAGTCTATTTACTTATTCTTTTATTTGTTATTGTTACTCTGGAAGCGACAGTTGGTTTTGTATCACAAAGTGATTTGCTTACTGCTACTCAGATGTATAAATACCCTGGCTTCGGTTATGTATTTCGTGCTGGAGGGGTCGCTAAGGATTCTAGTGCTTATGGCAGCCTTGTTTTTTTATTAGGGATCACTGCATTAATTGAGTTAAGGAAAATTAGAACAAATAATAGATTGTTGTCGTTTATTATAATTTCGTGTTTATTAATTAATATATATATATCTATGTCACGCAGCTTAATTGTATCTGTTGCATTATACTTTTTGATATATATATGCATGGAGAAAAAATCTCGAGTTAAATCATTTCTTATCCTAGGAGTAGTGTCTTTAGTTATTTTTATGTATGGACTTACAAATGAATATTTTTTATCATTTTTAAATAGATTGACAGGGGGAGAGCAAACAGACATCAGTTCCGGACGATTTTCAACTTGGGCTTCTTTGCTTGTACTTATTGCTGAAAATCCTATTTTCGGTGTAGGGTATAGATTAACTACAGAAAAATATAATTTAATACCTGACAATATGTTTCTTTCATTATTGGTAGAAACGGGAATAATTGGGTTTATATTATACTTTTCATTTTTAACCTGCTTGCTGATTTATGTTATAAAATATAATCGAGATAAGTTCCCATTGCTTGTGGCATATATATTCTCTGGCTTTTTTATCGACTCCGCTCTG | AB289652 | 99.80% |
